# Editorialets

**Published:** 1906-12

**Authors:** 


### Editorialets.

DR. GEORGE THOMAS PALMER, who for the past five years has edited *The Chicago Clinic* and *Pure Water Journal,* has voluntarily severed his connection with the Illinois State Board of Health, in which he held the position of Assistant Secretary, to devote his time and attention to the interests of his journal. For some years *The Clinic* has devoted considerable attention to climatology and mineral water therapy, being the only publication in the United States dealing with the latter subject. These special departments will be especially developed during the coming year. In his connection with the Illinois State Board of Health, Dr. Palmer has been actively engaged in sanitary and public health work, and a new department of State Medicine, edited by him, will be a feature in *The Clinic.*

---

DR. W. E. FITCH, editor of *Gaillard's*, a native North Carolinian, was a student at the medical department of the University of New York, where he attended his first course of lectures, graduating in medicine from the College of Physicians and Surgeons in 1891. Since his graduation his desire has been, some day, to locate in New York City. In 1901 he and the lamented I. N. Love formed a copartnership to practice medicine and to jointly unite their forces in medical journalism. With the expectation of the consummation of this association with Dr. Love, Dr. Fitch at once complied with all the laws and legal requirements of the New York statutes governing the practice of medicine, secured his license and had same registered in the New York county clerk's office in January, 1902, expecting to move to New York City in October of the same year, but the unexpected and untimely death of Dr. Love in June, 1902 for the time disconcerted his plans. Dr. Fitch has just associated himself with J. Adelphi Gottlieb, M. D., M. A., LL. D., of New York City, where they will have offices at No. 21 West Ninety-seventh street. Dr. Fitch and his family will live at the LaFayette, 320 Manhattan avenue, opposite Morning Side Park.

---

FOR THE CANCER PATIENT.—In keeping the cancer patient clean and wholesome, Therapogen will be found of great usefulness. In 3 to 5 per cent solution it kills all bacteria and removes all disagreeable odors. It moreover decreases the suppurative process, diminishes inflammation and makes the patient much more comfortable. In this special condition, therefore, experience would seem to indicate that Therapogen has a utility all its own.

---

$2500-$3000.—Unopposed country practice free to purchaser of fine residence, office, outbuildings and three acres of good land; 100 miles west of Houston. Collections, 98 per cent; $2000, one-half cash, balance to suit purchaser. Address, "Doctor," box 28, Route 3, La Grange, Texas.

---

IN ORDER to make the name of the laxative more fully descriptive of it, the California Fig Syrup Company has made an addition to the name, and in future the full name, which will be printed on the wrappers and labels of every bottle, will be "Syrup of Figs and

Elixir of Senna," which is what the remedy really is; its special excellence being due to the original method of obtaining the laxative principles of the senna and eliminating the griping and inert properties of the plant. We think that the new name will be all the more acceptable to physicians, as it describes the laxative more fully than the shorter name of Syrup of Figs.

---

Dr. Daniel's New Book, "The Strange Case of Dr. Bruno." See advertisement of Guarantee Publishing Company, under "Contents," last page.

---

For Sale.—$2500 unopposed country practice; nearest doctor ten miles. Collections 95 per cent; no boll weevil; good house, barn, office, private water system; many Germans. Price, $1600.
Dr. T. E. Boyer, Shive, Texas.

---

Deserved Compliment.—Dr. C. M. Rosser, of Dallas, one of the most distinguished physicians of the South, was made President of the "New Association of the Southwest" at its organization at Oklahoma City last month. See report of the meeting elsewhere in this issue. It will be observed that this organization is not in opposition or antagonistic to any existing association, but in harmony and coöperation with the great National Association.

---

For Sale.—A small, clean, up-to-date stock of drugs; a good business established; the store for sale or rent. Address Frank Ashley Drug Co., Rockett, Texas. H. W. Aldridge.

---

For the enlargement and betterment of the *Oklahoma Medical News-Journal.* Beginning with the January, 1907, issue, the *Oklahoma Medical News-Journal* will have a new editor, Y. E. Colville, B. S., M. D., of Chattanooga, Tenn. Dr. Colville has bought a half interest in the *Journal* and will devote his entire time to the editorial department, while Dr. Phelan will be the business manager. In this way the *Journal* will be greatly benefited and enlarged, and of greater value to the profession than heretofore.

---

Chloroform should not be administered too close to a gas jet or gas stove, as its vapors are thereby decomposed, forming products which, when inhaled by the patient, surgeon and assistants may give rise to disagreeable and even serious effects, such as neusea, vomiting, and pulmonary and renal irritation. — *International Journal of Surgery.*

